# Ecological Niche Modeling of Pathogenic *Leptospira* spp. and Risk Prediction of Canine Leptospirosis Occurrence in the Russian Federation

**DOI:** 10.3390/pathogens15070742

**Published:** 2026-07-15

**Authors:** Sergey V. Shcherbinin, Daria A. Bykovtseva, Natalia S. Bardina, Andrey V. Varkentin

**Affiliations:** All-Russian Research Institute for Animal Health, Vladimir 600901, Russia; bykovceva@arriah.ru (D.A.B.);

**Keywords:** leptospirosis, dogs, ecological niche modeling, MaxEnt, One Health, Russian Federation, spatial risk analysis

## Abstract

Leptospirosis remains a significant worldwide zoonotic threat, with domestic dogs playing a pivotal role in pathogen circulation across anthropogenic landscapes. This study aimed to assess the spatial distribution of ecological niches of pathogenic *Leptospira* spp. and conduct epizootic risk mapping within the Russian Federation. A two-stage modeling approach based on the maximum entropy algorithm (MaxEnt) was deployed. At the first stage, the abiotic model revealed the fundamental survival niche of the pathogen, identifying precipitation (700–1200 mm) and temperature (7–12 °C) as critical determinants. The accuracy of this model was AUC = 0.908 ± 0.007. At the second stage, the biotic model achieved a predictive accuracy of AUC = 0.980 ± 0.003, demonstrating that dog population density was the primary driver of risk, exhibiting a distinct threshold pattern (200–300 animals/km^2^). Furthermore, while rodent density remains the foundational ecological reservoir capable of independent transmission, the presence of additional domestic reservoirs, including livestock and dog populations, synergistically amplifies the transmission pathways, shaping the highest-risk zones within shared environments. These findings indicate distinct spatial risk boundaries within shared human–animal landscapes, suggesting that domestic dogs function as effective indicators for proactive zoonotic monitoring under the “One Health” framework.

## 1. Introduction

Leptospirosis is a widespread disease affecting domestic and wild animals, possessing significant zoonotic potential and caused by pathogenic spirochetes of the genus *Leptospira*. The distribution of these bacteria spans almost all continents, with the exception of Antarctica, encompassing diverse climatic zones and ecological niches. Epizootological data consistently confirm a high prevalence of leptospirosis in tropical and subtropical regions, where heavy precipitation and frequent flooding facilitate the contamination of water bodies and soil [1,2,3]. Natural foci of leptospirosis are ubiquitous, underscoring the high adaptability of *Leptospira* spp. and reflecting the stability of the ecological pathways driving their circulation [4,5].

Rodents (*Rodentia*) represent the primary reservoir and play a pivotal role in the dissemination of leptospires. In these hosts, the infection typically manifests in a subclinical form, which facilitates long-term shedding and continuous release of the bacteria into the environment. Beyond rodents, the host range extends to representatives of the orders *Carnivora*, *Didelphimorphia*, *Cetacea*, *Cingulata*, *Chiroptera*, *Afrosoricida*, as well as various reptiles and amphibians [6]. Among livestock, cattle, pigs, sheep, and goats contribute significantly to the emergence of outbreaks, wherein the disease may progress in clinical as well as in subclinical forms [7,8]. Dogs represent a major reservoir and a primary source of pathogenic leptospire transmission to humans. Their behavioral features, including frequent contact with soil and natural water bodies, facilitate environmental contamination. Furthermore, the growth of dog populations in urbanized areas contributes to the establishment of an endemic urban cycle of leptospirosis. Epidemiological data from the 1990s in Russia demonstrate a direct correlation between the expansion of stray dog populations, the deterioration of sanitary and hygienic conditions in cities, and the subsequent rise in canine leptospirosis cases. Notably, spikes in leptospirosis incidence recorded after floods and other extreme weather events further confirm the strong association between the disease and environmental factors [9].

The primary route of animal infection is through indirect contact via transmission factors, such as the urine of infected individuals and contaminated environmental objects. Animals with chronic leptospirosis are capable of long-term shedding of the pathogen due to its persistence in the kidneys and reproductive organs [10]. Despite the high prevalence of leptospires in tropical and subtropical regions, they are also periodically detected in Siberia and the Far East [4,11]. This evidence confirms that *Leptospira* spp. successfully adapt to diverse climatic conditions, leading to the formation of both natural and anthropurgic foci [12,13,14].

Currently, the diagnosis of leptospirosis remains a challenging task. *Leptospira* spp. cultivation requires specialized media and substantial time due to slow bacterial growth, while serological methods, such as the microscopic agglutination test (MAT), are frequently complicated by cross-reactivity between serovars. Molecular methods, such as PCR, offer higher sensitivity, but their applications remain limited in regions with insufficient laboratory infrastructure [15,16,17].

Due to underreporting, the true prevalence of leptospirosis among susceptible animals significantly exceeds officially recorded figures. This discrepancy poses a severe risk for the formation of persistent natural foci and threatens the sanitary and epidemiological well-being of the population [18,19]. Therefore, studying the dissemination of leptospires among animals is a critical step toward developing effective control and prevention measures [7,20,21].

The aim of this study was to evaluate the suitability of the Russian Federation territory for the establishment of environmental niches of pathogenic leptospires, as well as identify areas with the highest risk of canine leptospirosis occurrence. While traditional MaxEnt-based epidemiological studies establish a crucial foundation by mapping historical occurrence points against overlapping environmental variables [22,23,24], integrating broad eco-climatic indicators and localized host density indices into a single execution layer can sometimes complicate the isolation of specific risk drivers. To complement these established methodologies, our strategy utilizes an approach with segregated predictor groups that independently evaluates abiotic environmental boundaries and localized multi-host density matrices, thereby providing deeper insight into zoonotic risk evaluation.

## 2. Materials and Methods

### 2.1. Study Design

Two prognostic models were developed in the course of this study. The first model, an “abiotic” model, utilized environmental factors as predictors to reflect the potential suitability of landscape and climatic conditions (including climate, soil properties, and the presence of water bodies) for the survival and persistence of *Leptospira* spp. in the environment. The second model, a “biotic” model, incorporated data on the distribution of key reservoir hosts of the pathogen to assess the probability of the formation of anthropurgic foci and the registration of canine leptospirosis cases.

The abiotic model was based on data regarding the spatial distribution of pathogenic *Leptospira* spp. Meanwhile, the biotic model was based on recorded cases of animal leptospirosis caused by the species *Leptospira interrogans*, which represents the dominant species of pathogenic leptospires in the etiological structure of canine leptospirosis infection within the territory of the Russian Federation [4,7].

### 2.2. Study Area

**Figure 1:**
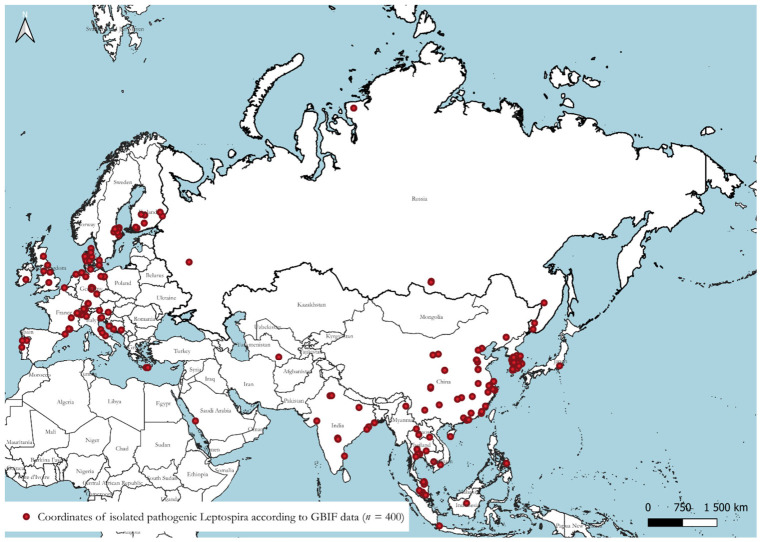
Geographic distribution of detection sites for pathogenic leptospires in environmental samples collected from 1977 to 2024.

The study area encompasses the Russian Federation and several neighboring Eurasian countries where pathogenic leptospires have previously been detected in environmental substrates (Figure 1).

### 2.3. Data Sources

Data on the detection of pathogenic *Leptospira* spp. in environmental samples were retrieved from the Global Biodiversity Information Facility (GBIF) database for the period from 1977 to 2024, which provides geographic coordinates (longitude and latitude) for sampling sites verified via laboratory testing [25,26]. Through this approach, coordinates for 400 *Leptospira* spp.-positive samples were obtained.

To gather information regarding the reservoir hosts of pathogenic leptospires, metadata from the international BioSample database was analyzed. The historical depth of the retrieved records spanned 109 years (1915–2024) [27]. A total of 1464 detection records for various serovars of the species *L. interrogans* were compiled (as of November 9, 2025). During data preprocessing, human-derived isolation records (*n* = 372), environmental samples (*n* = 21), and technological or duplicate entries lacking source information (*n* = 686) were excluded. The remaining dataset (*n* = 385) enabled the identification of specific reservoir host species (Table 1). Additionally, the global dataset GridScopeRodents was utilized to integrate data on the distribution of synanthropic rodents of the genera *Rattus*, *Apodemus*, and *Mus*, which represent the most commonly recorded carriers of leptospires [28].

Population density of the target species (*Canis familiaris*) was evaluated using generalized linear models (GLMs) with a negative binomial regression. This approach accounted for overdispersion in spatial abundance data. The WorldPop population density raster was utilized as the primary predictor (https://www.worldpop.org/) accessed on 21 February 2026 [29], after being transformed using the log(1 + x) formula to linearize the relationship and eliminate uncertainty associated with zero values. Model calibration was were performed based on independent statistical data from reports of the Center for the Study of Nutrition and Animal Welfare, IPSOS [30,31], and Rosstat (https://eng.rosstat.gov.ru/) accessed on 21 February 2026. Integrating these datasets enabled the establishment of a calculated dog population density coefficient (*k* = 0.1799) per capita, or approximately 1 dog per 6 people. Actual occurrence records of the species retrieved from the GBIF database were utilized for verification and predictive accuracy evaluation of the resulting model (*n* = 1346) [32], along with generated pseudo-absences (1:2). Validation using 10-fold cross-validation confirmed the high stability and reliability of the results: McFadden’s pseudo-coefficient of determination was *R*^2^ = 0.251 ± 0.002 and the Area Under the ROC Curve (*AUC*) reached 0.975. High True Skill Statistic (*TSS*) values = 0.866 ± 0.023 and *Kappa* = 0.843 ± 0.035 indicate the excellent discriminatory ability of the model and confirm the adequacy of using this anthropogenic factor for mapping risks associated with *Canis familiaris*. To analyze the spatial structure of the model residuals, a spatial autocorrelation analysis (Moran’s I test) was performed. The calculated Moran’s I was 0.1168 (*p* < 0.001), indicating the presence of a moderate positive autocorrelation, which is typical for large-scale environmental data; however, the model adequately approximates the global trend. The mean absolute error (*MAE*) was 0.397 ± 0.038, and the root mean square error (*RMSE*) was 0.878 ± 0.289, demonstrating the model’s robustness against local variations in density.

Data on the density of livestock (cattle, sheep, goats, horses, and pigs) was obtained from the FAO Gridded Livestock of the World ver. 4 (GLW4) information system [33].

The biotic model was based on data from the Ministry of Agriculture of the Russian Federation regarding the registration of animal leptospirosis outbreaks. The dataset covers the period from 2020 to 2026 and is derived from official quarantine regulatory acts [34]. It includes recorded cases of canine leptospirosis (*n* = 33), cattle (*n* = 32), small ruminants (*n* = 3), horses (*n* = 14) and pigs (*n* = 6).

### 2.4. Ecological Niche Modeling

Ecological niche modeling of pathogenic leptospires and the subsequent risk assessment of canine leptospirosis cases performed using the maximum entropy algorithm (MaxEnt). MaxEnt performs predictive modeling of the geographic distribution of species based on environmental conditions at their known occurrence sites. Since the algorithm estimates the target probability distribution, it is equally applicable for modeling the spatial distribution and transmission risk of infectious diseases [35]. A comprehensive set of climatic, landscape and environmental factors determining the survival and persistence of leptospiras in the environment was selected as predictors (Table 2). The relevance of these variables is well-supported by similar peer-reviewed studies and comprehensive reviews [22,23,24,36,37,38,39]. To ensure spatial data consistency, all raster layers were projected to a common coordinate system and resampled to a uniform spatial resolution of 1 × 1 km.

### 2.5. Model Construction and Validation

A preliminary Pearson correlation analysis was performed, and the variance inflation factor (VIF) was calculated to minimize multicollinearity among predictors. Variables demonstrating a high degree of interdependence were excluded from subsequent analysis, which improved model interpretability and minimized the risk of overfitting. For model construction and validation, a dataset of pathogenic leptospire occurrence sites and disease cases (presence data) and a set of predictors (Table 2) were used. In both models, the occurrence data were split into training and testing datasets in a 70/30% ratio. For the first model, the regularization multiplier (RM) was kept at its default value (*RM* = 1.0). For the second model, due to the limited volume of data on the epizootic situation (*n* = 88), the RM value increased to 3.0 to reduce potential model overfitting and smooth the feature classes. Background points (*n* = 5000) for both models were generated randomly across the entire study area. Modeling was performed using the bootstrap method with 10 iterations, with occurrence data randomly selected for each iteration. The resulting maps contained mean probability values (ranging from 0 to 1), which were classified using the Jenks natural breaks method to visualize areas with distinct risk levels. The predictive accuracy of the models was assessed using the Area Under the Curve (AUC). AUC values exceeding 0.8 were considered indicative of “high performance”, values between 0.7 and 0.8 were considered “good performance”, while an AUC below 0.7 indicated a lack of predictive ability [45]. To assess the importance of variables, the jackknife test was used: each variable was alternately included in and excluded from the model, after which the estimated gain associated with the addition of that specific variable was compared.

### 2.6. Software

Mapping and spatial data processing were performed using QGIS 3.44.7 [46]. The maximum entropy model was fitted using MaxEnt software version 3.4.4 [47]. All stages of data analysis, including execution of the MaxEnt algorithm and subsequent post-processing of the modeling results, were carried out within the RStudio statistical programming environment (version 2026.01.1+403) utilizing R software version 4.5.2.

## 3. Results

**Figure 2:**
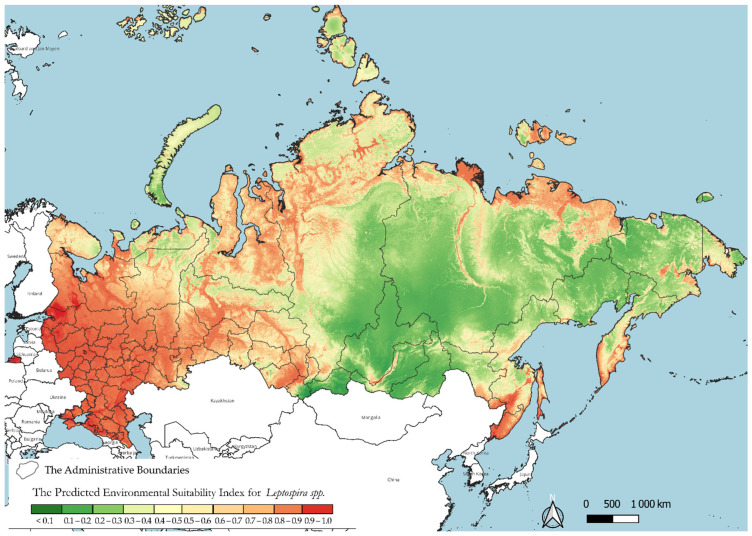
Predicted environmental suitability of the Russian Federation for the survival and persistence of pathogenic leptospires in soil and water.

The abiotic model, trained on environmental and climatic data using 5000 background points and 400 occurrence sites, visualized the environmental suitability of the Russian Federation for the survival and persistence of pathogenic *Leptospira* spp. (Figure 2).

**Figure 3:**
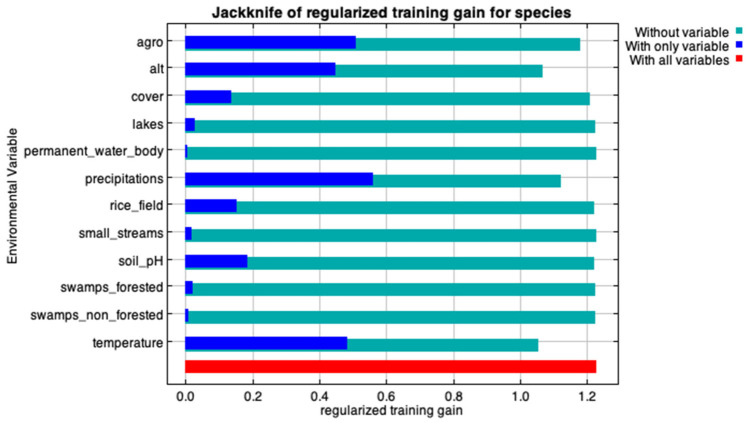
Results of the jackknife test for assessing the relative contribution of variables used to determine the ecological niches of pathogenic leptospires in Russia.

According to the constructed model (Figure 2), areas with high predicted environmental suitability for pathogenic *Leptospira* spp. (*HSI* = 0.7–1.0) are concentrated in the North Caucasus Federal District, the left-bank regions of the Dnieper, the western and central areas of the East European Plain, the floodplains of Western Siberia, the Russian Arctic regions, and the southern Far East. A high level of suitability is also predicted in Kaliningrad Oblast. The model demonstrated high predictive accuracy, with an AUC = 0.908 ± 0.007; *p* < 0.001. The variables contributing most to the final model were precipitation amount, temperature regime, elevation above sea level, and the proportion of cultivated land. Variables reflecting land cover type, the presence of rice paddies, and soil pH were of minor importance for the model (Figure 3).

The initial set of predictors for the first model included a soil moisture indicator; however, based on the correlation analysis results, this layer was excluded due to a high correlation coefficient with the soil pH layer *r* = −0.72 ± 0.008; *p* <0.001). Subsequent verification using the jackknife method showed that predictors characterizing water bodies in Russia do not contribute significantly to the model, as the relevant information is duplicated by the precipitation variable (Figure 3).

**Figure 4:**
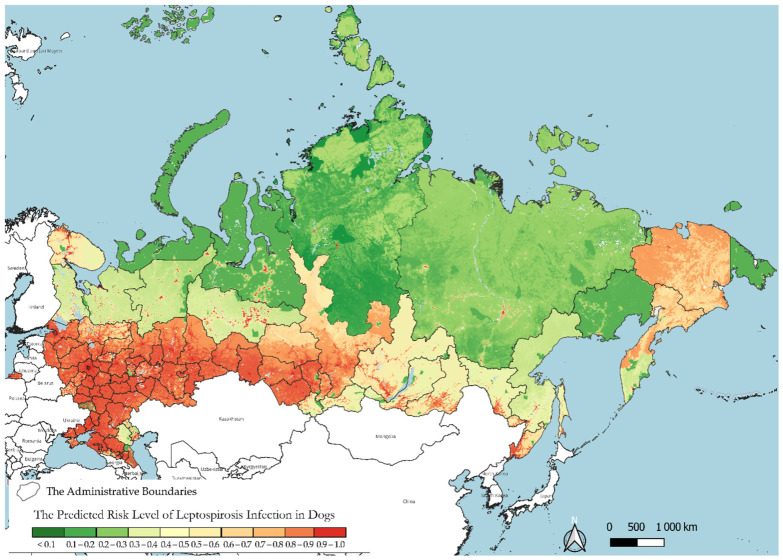
Predicted areas with a high risk of canine leptospirosis within the Russian Federation.

The second model visualized areas with the highest risk of canine leptospirosis cases within the Russian Federation (Figure 4).

As shown in Figure 4, areas with a high risk of canine leptospirosis infection have been identified across the Russian Federation (R = 0.7–1.0). They form a continuous range within the Northwestern, Central, Volga, and Southern Federal Districts, and also cover almost the entire North Caucasus Federal District. A characteristic feature of this model is a high spatial heterogeneity within the Siberian and Far Eastern Federal Districts. Here, high-risk areas are localized mainly in the southern border regions, corresponding to the most urbanized areas. In most administrative centers of these regions, risk values reach maximum levels (0.9–1.0).

For the construction of the biotic model, the a priori set of predictors included a layer of leptospire habitat suitability obtained at the first stage of modeling, as well as the “agro” variable layer used to differentiate between rural and urban areas. However, during correlation analysis, the habitat suitability factor was excluded due to high interdependence with the rodent population density (*r* = 0.764 ± 0.0002; *p* < 0.001). The “agro” variable was also removed from the final model due to significant correlation with cattle population density (*r* = 0.657 ± 0.0007; *p* < 0.001) and the absence of a pronounced relationship with the population characteristics of the target species. In addition, goat population density, which demonstrated a high positive correlation with horse population density, was excluded from the analysis (*r* = 0.77 ± 0.038; *p* < 0.001).

**Figure 5:**
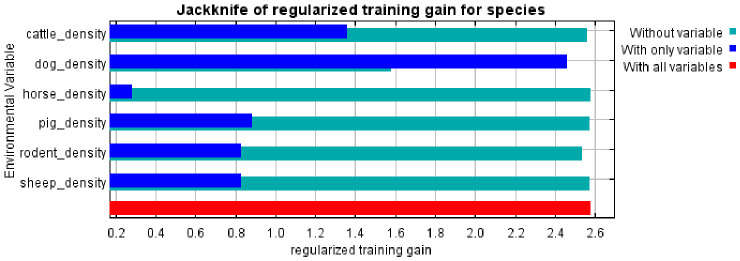
Results of the jackknife test for assessing the relative contribution of variables used to predict cases of canine leptospirosis in Russia.

The biotic model showed high predictive accuracy: AUC = 0.980 ± 0.003; *p* < 0.001. The most important predictive variables for risk realization were dog and cattle population densities; meanwhile, rodent density acts as a widespread baseline reservoir, whose potential is synergistically enhanced by the presence of livestock and domestic hosts (Figure 5).

**Figure 6:**
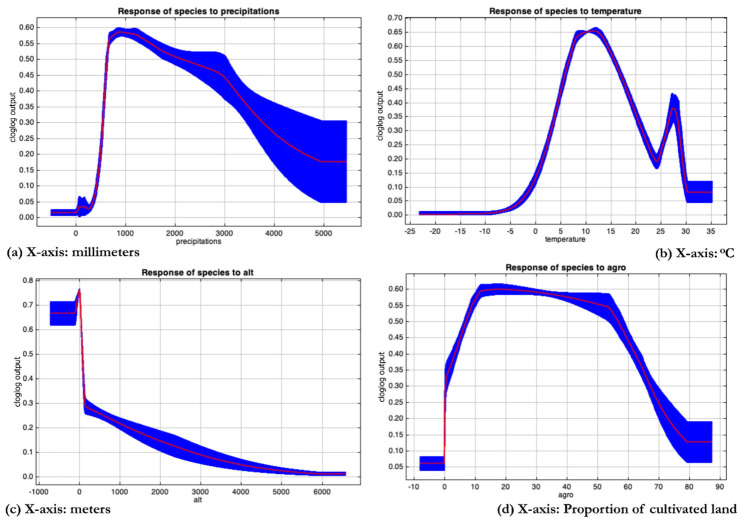
**Response curves of the significant variables in the abiotic model:** (**a**) mean annual precipitation; (**b**) mean annual air temperature; (**c**) elevation above sea level (altitude); (**d**) percentage of cultivated land. The red lines show the mean trends across 10 model replicates, while the blue areas indicate the standard deviation limits. The *Y*-axis represents the relative habitat suitability calculated using only the specific variable. Measurement units for the *X*-axes are provided in the respective graph labels and Table 2.

**Figure 7:**
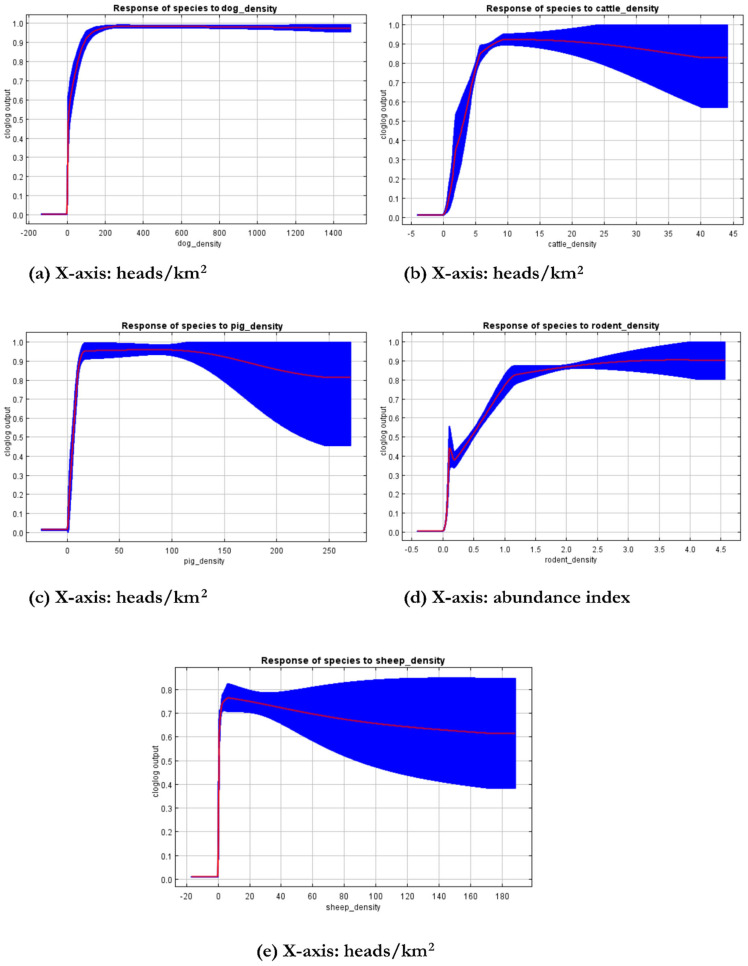
**Response curves of the significant variables in the biotic model:** (**a**) dog population density; (**b**) cattle population density; (**c**) pig population density; (**d**) rodent population density; (**e**) sheep population density. The red lines show the mean trends across 10 model replicates, while the blue areas indicate the standard deviation limits. The *Y*-axis represents the relative probability of infection calculated using only the specific variable. Measurement units for the *X*-axes are provided in the respective graph labels and Table 2.

Figure 6 and Figure 7 display the response curves for the most significant variables in the abiotic and biotic models, respectively.

## 4. Discussion

This study identified the most suitable habitats within the Russian Federation for the survival and persistence of leptospires in the environment, as well as the potential risk areas for canine leptospirosis occurrences.

Modeling confirmed that precipitation is the key limiting factor for *Leptospira* spp. in the Russian Federation, with an ecological optimum of 700–1200 mm (Figure 6a). The decline in habitat suitability above this threshold is likely due to the degradation of habitat conditions for the primary reservoirs (rodents) caused by waterlogging [48]. It was expected that the “rice_field” variable would contribute more substantially to the model, given the development of rice cultivation in southern Russia. However, this parameter demonstrates higher sensitivity in other agroclimatic conditions, for example, in the Asia-Pacific region, where rice cultivation occupies a significant share of crop production [49].

The response curve for the temperature factor (Figure 6b) exhibits a bimodal shape, reflecting the high adaptability of leptospires to different climatic zones.

The primary optimum (7–12 °C) corresponds to temperate latitudes, where low evaporation rates help maintain soil moisture. The increase in suitability observed at 0–5 °C is consistent with published data on bacterial detection in the Arctic [23,36,50].

The secondary peak (27–28 °C) reflects the period of active leptospire multiplication in stagnant water bodies. This interval coincides with seasonal peaks of disease incidence, periods of agricultural field work, and high recreational pressure on water bodies [20,51,52].

The model confirms the strong association of the pathogen with lowland areas (Figure 6c). Maximum suitability was recorded at elevations close to sea level and in low-lying depressions, where slow runoff contributes to the formation of stagnant water bodies [33]. When rising above 200 m, the predicted risk drops sharply. The high suitability of the North Caucasus territories (Figure 2) is explained by the synergy of several factors: a temperature optimum (27–28 °C), a developed river network (Kuban, Terek), the orographic effect, and high livestock population density. This is consistent with data on the high risk of leptospirosis in mountainous and foothill regions of the world [39,53].

Analysis of the “agro” variable (Figure 6d) revealed a nonlinear relationship. Maximum suitability is achieved at 15–50% of cultivated land. Moderate landscape development, the creation of irrigation canals, and moistening of arable land create ideal conditions for spirochete circulation. At high levels of cultivation (above 60%), suitability decreases, which may be explained by the destruction of rodent shelters or the intensive use of agrochemicals [33].

The versatility of the abiotic model is confirmed by the presence of high-risk areas (*HSI* > 0.7) in contrasting environmental conditions: from the low-lying Kaliningrad Oblast with its maritime climate to the Far East, where the monsoon moisture regime plays a key role [48,50].

The modeling results explain the low predictive power of open water bodies compared to soil moisture. This confirms the hypothesis that the soil substrate serves as the primary habitat for *Leptospira* spp., ensuring their long-term survival. Contamination of open water bodies (lakes, ponds, streams) is predominantly secondary in nature. It occurs during periods of heavy rainfall or floods due to the washing out of bacteria from the surface layers of soil. Thus, the presence of water bodies alone is not a determining factor of habitat suitability without considering the hydrological regime of soils [2].

Analysis of the response curves (Figure 7) revealed key patterns for the variables of the biotic model.

The “dog_density” factor (Figure 7a) demonstrates a pronounced logistic relationship. The risk increases sharply at minimal density values, reaching a saturation threshold at 200–300 animals/km^2^. The curve reaching a plateau indicates that once this density is achieved, conditions for leptospire circulation become maximally favorable, and further population growth no longer increases the probability of infection.

The variables “cattle_density” and “pig_density” (Figure 7b,c) are characterized by a sharp increase in risk at minimal livestock density, followed by a decline at higher values. The decrease in risk at cattle densities above 20 heads/km^2^ and pig densities above 120–150 heads/km^2^ is explained by the transition to closed-type industrial livestock farming. Strict biosecurity measures at such facilities minimize animal contact with the external environment, thereby limiting bacterial circulation.

The “rodent_density” variable demonstrates a sharp increase in infection risk (up to 0.5) at a minimal population index of 0.1, indicating that rodents represent the fundamental primary reservoir capable of independent pathogen transmission. However, the model suggests that while rodents sustain this foundational baseline circulation of leptospires, the presence of additional domestic hosts, specifically livestock and dogs, acts to synergistically amplify the transmission pathways, scaling up the risk within shared environments. The “sheep_density” factor is significant mainly in traditional sheep farming regions (southern Russia). High model uncertainty at high population density values suggests that sheep are not a stable global predictor.

The primary spatial influence of dog population density highlights the potential risk of pathogen transmission within shared human–animal environments. Within the framework of the “One Health” concept, since dogs live in close proximity to humans and can shed leptospires, the generated risk maps (Figure 4) should be considered as predictive early-warning tools to evaluate potential zoonotic exposure. Dogs, being highly susceptible, act as sentinel animals. The identified high-risk areas should become priority zones for strengthening veterinary surveillance and mandatory vaccination, conducting educational work among the population, and integrating the models into the public health system to transition toward proactive risk management of zoonoses.

## 5. Limitations of the Study

Despite the high accuracy of the models, the study carries several limitations that should be taken into account when interpreting the results:

1. The use of a single coefficient for dog density (*k* = 0.1799) represents a necessary simplification for the national scale. In reality, because this parameter functions as a modeled statistical proxy derived from regional datasets (such as Rosstat and market surveys) rather than a directly observed nationwide census, it carries inherent limitations. This indicator may vary depending on the level of urbanization and regional cultural characteristics, and localized inaccuracies within the underlying source matrices could introduce unrecognized spatial uncertainty into the final predictive output.
2. The spatial structure of case registrations is biased toward regions with developed veterinary infrastructure. This may lead to underreporting of disease incidence in remote and sparsely populated areas.
3. Leptospirosis was considered a single nosological entity. The lack of differentiation by serovars (Canicola, Icterohaemorrhagiae, Grippotyphosa, and others) limits the assessment of niche specificity for individual etiological agents.
4. Another limitation of this model is the exclusion of dynamic factors such as regional vaccination coverage for dogs and the changing intensity of veterinary surveillance. These parameters vary from year to year, and there are no standardized national geospatial datasets available for them. While these anthropogenic factors may influence local epidemiological patterns, it is not feasible to include them in a long-term macro-regional spatial model.
5. Additionally, for the utilization of multi-source historical databases, while these platforms aggregate large amounts of epidemiological data, their completeness and correctness depend entirely on the primary contributors who enter the records. This is an inherent baseline assumption that must be taken into account when interpreting the final risk maps.

## 6. Conclusions

This study establishes a two-stage framework for evaluating leptospirosis risk. Abiotic factors form the fundamental survival niche of the bacteria, while the presence of rodents ensures the enzootic nature of leptospirosis. However, dog population density serves as the key indicator of epizootic risk realization. The high significance of this predictor is explained by the multifunctional role of dogs in anthropogenic landscapes: performing herding and guarding functions, they facilitate contact between wild reservoirs and farm animals, while their status as companion animals determines their close integration into the human environment. Thus, a high dog density in areas with favorable abiotic conditions serves as a reliable spatial proxy for identifying zones where closer integration of veterinary and public health surveillance is needed to mitigate zoonotic risks within the framework of the “One Health” concept.

## Figures and Tables

**Table 1:** Host range of *L. interrogans* based on BioSample data collected from 1915 to 2024 (the number of positive records is indicated in parentheses) [28].

Rodents and Insectivores	Domestic Carnivores	Farm Animals	Wild Terrestrial Mammals	Marine Mammals
*Rattus norvegicus* (122)	*Canis familiaris* (70)	*Sus scrofa domesticus* (36)	*Sus scrofa* (8)	*Zalophus wollebaeki* (16)
*Apodemus speciosus* (20)	*Felis catus* (4)	*Bos taurus* (23)	*Procyon lotor* (7)	*Zalophus californianus* (2)
*Rattus rattus* (11)		*Equus caballus* (9)	*Urva auropunctata* (7)	
*Mus musculus* (9)		*Ovis* sp. (4)	*Meles* sp. (5)	
*Apodemus agrarius* (7)		*Capra* sp. (1)	*Mephitis mephitis* (2)	
*Rattus flavipectus* (3)			*Erinaceus europaeus* (2)	
*Alexandromys fortis* (2)			*Pentalagus furnessi* (1)	
*Apodemus flavicollis* (1)			*Cervus elaphus* (1)	
*Apodemus chevrieri* (1)			*Canis latrans* (1)	
*Rattus losea* (1)			*Canis lupus* (1)	
*Mesocricetus auratus* (1)			*Vulpes vulpes* (1)	
*Microtus arvalis* (1)			*Erinaceus roumanicus* (1)	
*Sorex araneus* (1)				
*Mus caroli* (1)				
*Cavia aperea* (1)				
*Hydrochoerus hydrochaeris* (1)				
Total number of affected animals by type				
183	74	73	37	18

**Table 2:** Predictor variables characterizing the ecological and geographical conditions used for ecological niche modeling.

Variable Name	Variable Value	Factor Type	Unit	Source
temperature	annual mean air temperature	climatic	°C	https://www.worldclim.org/ [40]
precipitations	mean annual precipitation	climatic	mm	https://www.worldclim.org/ [40]
lakes	presence of lakes	hydrological	%	https://www.hydrosheds.org/ [41]
swamps_forested	presence of forest swamps	hydrological	%	https://www.hydrosheds.org/ [41]
swamps_non_forested	presence of non-forest swamps	hydrological	%	https://www.hydrosheds.org/ [41]
permanent_water_body	presence of permanent water bodies	hydrological	%	https://www.hydrosheds.org/ [41]
small_streams	presence of streams	hydrological	%	https://www.hydrosheds.org/ [41]
rice_field	presence of rice paddies	hydrological	%	https://www.hydrosheds.org/ [41]
soil_pH	soil pH at 0 cm depth	landscape	рН × 10	https://doi.org/10.1371/journal.pone.0169748 [42]
alt	elevation above sea level	landscape	meters	https://www.worldclim.org/ [40]
cover	land cover types	landscape	category	https://due.esrin.esa.int/ [43]
agro	proportion of cultivated land	landscape	%	https://www.fao.org/soils-portal/ [44]
cattle_density	cattle population density	population	animals per km^2^	https://www.fao.org/ [33]
pig_density	pig population density	population	animals per km^2^	https://www.fao.org/ [33]
horse_density	horse population density	population	animals per km^2^	https://www.fao.org/ [33]
sheep_density	sheep population density	population	animals per km^2^	https://www.fao.org/ [33]
rodent_density	rodent population density index	population	density index	https://doi.org/10.1038/s41597-025-05793-0 [28]
dog_density	dog population density	population	animals per km^2^	Developed regression model (current study)

## Data Availability

The datasets generated and/or analyzed during the current study are available from the corresponding author upon reasonable request.
